# Transitions in general practice training: quantifying epidemiological variation in trainees’ experiences and clinical behaviours

**DOI:** 10.1186/s12909-022-03178-0

**Published:** 2022-02-23

**Authors:** Michael Tran, Susan Wearne, Amanda Tapley, Alison Fielding, Andrew Davey, Mieke van Driel, Elizabeth Holliday, Jean Ball, Kristen FitzGerald, Neil Spike, Parker Magin

**Affiliations:** 1 School of Medicine, Western Sydney University, Narellan Road & Gilchrist Drive, Campbelltown, NSW 2560 Australia; 2 Academic Unit of General Practice, Australian National University, The Canberra Hospital, Yamba Drive Garran, Canberra, ACT 2605 Australia; 3 School of Medicine and Public Health, University of Newcastle, University Drive, NSW 2308 Callaghan, Newcastle, Australia; 4 GP Synergy, Regional Training Organisation (RTO), NSW & ACT Research and Evaluation Unit, 20 McIntosh Drive, Mayfield West, NSW 2304 Australia; 5 Primary Care Clinical Unit, Faculty of Medicine, University of Queensland, Level 8, Health Sciences Building, Royal Brisbane & Women’s Hospital, Brisbane, QLD 4029 Australia; 6 Clinical Research Design and Statistical Support Unit (CReDITSS), Hunter Medical Research Institute (HMRI), Lot 1, Kookaburra Cct, New Lambton Heights, NSW 2305 Australia; 7 School of Medicine, University of Tasmania, 17 Liverpool Street, TAS 7000 Hobart, Australia; 8 General Practice Training Tasmania (GPPT), Regional Training Organisation (RTO), Level 3, RACT House, 179 Murray Street, Hobart, TAS 7000 Australia; 9 Department of General Practice and Primary Health Care, University of Melbourne, 200 Berkeley Street Carlton, Victoria, 3053 Australia; 10 Eastern Victoria General Practice Training (EVGPT), Regional Training Organisation (RTO), 15 Cato Street, Hawthorn, VIC 3122 Australia; 11 Faculty of Medicine, Nursing and Health Sciences, School of Rural Health, Monash University, Northways Road, Churchill, VIC 3842 Australia

**Keywords:** Education, Medical, Graduate, Family practice, General practice, Primary health care, In-practice experience, Change management, Social theory

## Abstract

**Background:**

General Practice training in Australia is delivered through the apprenticeship model. General Practice supervisors support trainees transitioning from hospital-based work towards competent independent community-based practice. The timing and manner in which support should be provided is still not well understood. This study aimed to establish the variation in clinical and educational experiences and behaviours, and location, of general practice trainees’ consultations by stage of their vocational training. It was hypothesised that change is greater in earlier stages of training.

**Methods:**

A cross-sectional analysis of data (2010–2018) from the Registrar Clinical Encounters in Training (ReCEnT) study, an ongoing cohort study of Australian GP registrars’ in-consultation clinical and educational experience and behaviours. Multinomial logistic regression assessed the association of demographic, educational, and clinical factors in different stages of training. The outcome factor was the training term.

**Results:**

Two thousand four hundred sixteen registrars contributed data for 321,414 patient consultations. For several important variables (seeing patients with chronic disease; new patients; seeking in-consultation information or assistance; ordering pathology and imaging; and working in a small or regional practice), odds ratios were considerably greater for comparisons of Term 1 and 3, relative to comparisons of Term 2 and 3.

**Conclusion:**

Differences experienced in demographic, clinical and educational factors are significantly more pronounced earlier in registrars’ training. This finding has educational and training implications with respect to resource allocation, trainee supervision and curriculum design. Sociocultural learning theory enables an understanding of the impact of transitions on, and how to support, general practice trainees and supervisors.

## Background

General practice (family medicine) is a specialty which allows healthcare systems to produce high quality, equitable and efficient care [1]. Inherent in general practice is the management of a high proportion of undifferentiated illnesses and the inevitable uncertainty that permeates decision-making [2]. Internationally, various training programs exist to prepare trainees for the change from hospital-based work to general practice work and learning [3]. Successfully navigating this change is important to reduce negative consequences including burnout and intolerance of clinical uncertainty [4].

Australian general practice specialist vocational training (hereafter, ‘GP training’), like many medical vocational training programs, is based on the clinical apprenticeship-like model. Registrars (trainees) work independently alongside experienced general practitioner (GP) supervisors [5]. This model provides exposure to a wide-ranging set of patient demographics and clinical presentations. After graduating from medical school, GP registrars will complete a minimum of 24 months of hospital-based training, 18 months of general practice placements and 6 further months of extended skills training (which may be undertaken in general practice or in hospital-based training). There are shared experiences between Australian GP training and post-graduate medical specialist training programs internationally [6].

The transition from medical student to junior doctor entails individuals coping with new responsibilities, tasks and expectancies [7, 8]. This can initiate a period of rapid personal development with development of robust coping strategies [9], but can also leave practitioners prone to burnout [10]. A similarly momentous transition occurs between hospital-based work and general practice training, where registrars are required to adopt a high level of independence within an apprenticeship model of working, with similar risks for adverse outcomes [11]. What is not well-understood is the rate and impact of the transition that occurs within general practice training and beyond this in independent practice.

Differences in clinical and learning behaviour between early and later stages of GP training have previously been found in cross-sectional analyses for various outcomes including the management of chronic disease [12]; information-seeking by trainees [13]; use of resources and information within a consultation [14]; generation of learning goals [15]; anxiety due to clinical uncertainty [2]; location (rurality) of practice [16]; and use of pathology ordering [17]. In identifying these differences, a better understanding is generated of how the individual skills that comprise clinical competence in independent practice change with progression through training. Previous research has not systematically assessed exactly when these differences occur during training. These aforementioned factors in clinical experience and behaviours have not previously been synthesised in a single analysis by stage of training. Understanding the timing of these changes and where transitions are most keenly experienced has important general practice workforce, training, and educational implications. It would help optimise educational resource allocation and trainee and supervisor support. These findings could be applied to other training programs with an apprenticeship-like model of training.

In this exploratory epidemiological study, we sought to establish variation in clinical and learning content, and location, of trainee consultations by stage (training term) of training. We hypothesised, given previous individual findings using training term as an independent factor, that changes in demographic, clinical and educational variables would be greatest earlier in training.

## Methods

This analysis was conducted within the Registrar Clinical Encounters in Training (ReCEnT) project.

### ReCEnT

ReCEnT is an ongoing cohort study of GP registrars’ in-consultation clinical and educational experience and behaviours. It was conducted in five training organizations in five Australian states from 2010 to 2015, and in three training organizations in three states and a territory from 2016 (following a major reorganization of the national GP training program). The study participants are Australian College of Rural and Remote Medicine and Royal Australian College of General Practitioners registrars in general practice training terms.

Registrars complete data collection, once in each of three six-month (full-time equivalent) training terms, as a routine component of their training program [18, 19]. They may also provide consent for their data to be used for research purposes.

Data collection includes registrars’ demographic data, and practice characteristics. Details of 60 consecutive clinical consultations per term via a paper-based case report form are recorded. Only office-based consultations are recorded. The consultation data includes patient demographics, diagnoses and problems managed, investigations, management and educational training aspects. The detailed methodology is presented elsewhere [18].

### Outcome

The outcome factor was stage of registrar training (Term 1, 2, or 3).

### Independent variables

Independent variables related to the registrar, practice, patient, consultation, and outcomes of the consultation. See Table 2 for individual independent variables included in analyses.

### Statistical analysis

A cross-sectional analysis of 18 rounds of data (2010 – 2018) from the longitudinal ReCEnT study, was conducted at the level of registrar-round and at the level of the consultation. The proportion of registrar-rounds and consultations at each training stage were calculated with 95% confidence intervals adjusted for repeated measures within registrars.

Descriptive statistics included frequencies with percent for categorical variables and mean with standard deviation for continuous variables. The frequencies of categorical variables were compared between outcome categories using Chi-squared tests for all variables, except when Fisher’s exact test was used (due to an expected count less than 5 in 25% or more cells). For continuous variables, means were compared using a t-test.

Multinomial logistic regression was used within the generalised estimating equations (GEE) framework to account for repeated measures within registrars. The reference category of the nominal outcome was specified as Term 3 for all analyses.

Univariate analyses were conducted on each covariate, with the outcome. Covariates with a univariate *p*-value < 0.20 were considered for inclusion in the multiple regression model.

Once the model with all significant covariates was fitted, model reduction was assessed. Covariates which were no longer significant (at *p* < 0.2) in the multivariable model were tested for removal from the model. If removal of the covariate did not substantively change the resulting model, it was removed from the final model.

The regressions modelled the log-odds that registrars were in Term 1 or Term 2, compared to Term 3, as a function of independent variables.

To investigate the research question regarding how registrars’ clinical behaviours, educational experience and training location differed by stage of training, four regression models were built, to sequentially assess associations of different variable types with the outcome.

In the first model, the differences in registrars and their training practice over stages of training were assessed, by including practice and registrar independent variables in the regression model. Analysis was at the level of registrar-round.

The second model examined patient differences across registrar training stages, by including all the above variables, together with patient variables in the regression model. Analysis was at the consultation level.

Similarly, to examine consultation and consultation-outcome differences across training stages, the above processes were repeated in third and fourth regression models, both analysed at the consultation level.

The rationale for this sequential approach was that we sought to establish associations of registrars’ practice location with training term (model 1) adjusted for registrar and practice factors, but not for patient or consultation variables which are not relevant to practice location allocation. Similarly, we sought to establish patient associations with registrar term (model 2) adjusted for registrar and practice factors, but not for consultation or consultation-outcome which are not relevant to the registrar ‘seeing’/being consulted by the patient. Similar logic applied for models 3 and 4.

Effects were expressed as odds ratios (ORs) with 95% CI. Significance was declared at the conventional 0.05 level, with the magnitude and precision of effect estimates also used to interpret results. Analyses were programmed using STATA 14.0 and SAS V9.4.

### Ethics approval

University of Newcastle Human Research Ethics Committee, Reference H-2009–0323.

## Results

Data collection included 18 rounds of ReCEnT data collection (2010–2018), including 2416 registrars (response rate 94.9%), 5798 registrar-rounds, and 321,414 registrar-patient consultations. Registrar and practice characteristics are presented in Table 1.

**Table 1:** Characteristics of participating registrars and their practice

Registrar variables (*n* = 2416)		n (%)
Registrar gender	Female	1496 (62.3)
Qualified as doctor overseas	Yes	472 (19.7)
Registrar round/practice variables (*n* = 5798)		
Registrar age (years)	Mean ± SD	32.6 ± 6.3
Registrar works Part-time	Yes	1509 (26.4)
Worked at practice before	Yes	1258 (22.0)
Registrar training term	Term 1	2294 (39.6)
	Term 2	2100 (36.2)
	Term 3	1404 (24.2)
Practice rurality/urbanicity	Major city	3444 (60.2)
	Inner regional	1501 (26.4)
	Outer regional / remote / very remote	765 (13.4)
Practice location SES status (SEIFA index)	Mean ± SD	5.5 ± 2.8
Practice routinely bulk bills	Yes	1509 (26.4)
No. GPs (FTE) working at the practice	1–4	2071 (37.8)
	5 +	3413 (62.2)

Term 1 registrars contributed 39.6% [95% CI: 39.1–40.1] of registrars-rounds (*n* = 2294); Term 2 registrars 36.2% [95% CI: 35.8–36.6] (*n* = 2100); and Term 3 24.2% (95%CI 23.6–24.8] (*n* = 1,404]. The corresponding percentages for patient consultations were for Term 1, 40.3% (95%CI: 39.8–40.9] (*n* = 129,578); Term 2, 35.9% (95% CI: 35.4–36.3] (*n* = 115,314); and Term 3, 23.8% (95%CI: 23.2–24.5] (*n* = 76,522).

Characteristics associated with stage of training are presented in Table 2, with univariate and multivariable regression models presented in Table 3.

**Table 2:** Characteristics associated with stage of training

				**Registrar term**			
**Factor group**	**Variable**	**Class**	**Term 1**		**Term 2**	**Term 3**	**p**
**Registrar round level (*n* = 5798)**							
Registrar factors	Registrar gender	Male	871 (38%)		834 (40%)	538 (38%)	< 0.001
		Female	1415 (62%)		1260 (60%)	863 (62%)	
	Registrar Full Time or Part Time	Part-time	467 (21%)		367 (18%)	393 (30%)	< 0.0001
		Full-time	1737 (79%)		1618 (82%)	914 (70%)	
	Qualified as doctor in Australia	No	440 (19%)		384 (18%)	247 (18%)	0.0526
		Yes	1845 (81%)		1703 (82%)	1147 (82%)	
	Registrar age	mean (SD)	32 (6)		33 (6)	33 (6)	< 0.0001
Practice factors	Practice size	Small	913 (41%)		706 (36%)	452 (35%)	< 0.0001
		Large	1292 (59%)		1276 (64%)	845 (65%)	
	Practice routinely bulk bills	No	1639 (72%)		1505 (73%)	1068 (77%)	0.0016
		Yes	637 (28%)		552 (27%)	320 (23%)	
	Rurality	Major city	1334 (59%)		1243 (61%)	867 (62%)	< 0.0001
		Inner regional	607 (27%)		528 (26%)	375 (27%)	
		Outer regional / remote / very remote	336 (15%)		279 (14%)	150 (11%)	
	Geographic Region	Region 1	513 (22%)		487 (23%)	381 (27%)	< 0.0001
		Region 2	153 (7%)		159 (8%)	104 (7%)	
		Region 3	238 (10%)		224 (11%)	187 (13%)	
		Region 4	779 (34%)		722 (34%)	556 (40%)	
		Region 5	64 (3%)		59 (3%)	12 (0.9%)	
		Region 6	547 (24%)		449 (21%)	164 (12%)	
	SEIFA index	mean (SD)	5 (3)		6 (3)	6 (3)	< 0.0001
**Consultation level (*n* = 321,414)**							
Patient factors	Patient age group	0–14	21,724 (17%)		20,106 (18%)	13,183 (17%)	0.0132
		15–34	35,048 (28%)		30,839 (27%)	21,018 (28%)	
		35–64	47,947 (38%)		42,302 (37%)	27,760 (37%)	
		65 +	22,657 (18%)		20,431 (18%)	13,488 (18%)	
	Patient gender	Male	50,206 (40%)		44,559 (40%)	29,164 (39%)	0.0202
		Female	75,694 (60%)		68,201 (60%)	45,806 (61%)	
	Aboriginal and/or Torres Strait Islander	No	118,669 (98%)		104,752 (98%)	71,361 (99%)	0.0348
		Yes	2033 (2%)		1848 (2%)	1019 (1%)	
	Non-English speaking background	No	109,919 (90%)		98,601 (92%)	67,877 (93%)	< 0.0001
		Yes	11,696 (10%)		8754 (8%)	4909 (7%)	
	Patient/practice status	Existing patient	43,921 (35%)		49,638 (44%)	32,257 (43%)	< 0.0001
		New to registrar	72,176 (57%)		55,169 (49%)	37,457 (50%)	
		New to practice	10,200 (8%)		7943 (7%)	5446 (7%)	
Consultation factors	Chronic problem	No	95,686 (74%)		84,822 (74%)	55,943 (73%)	0.1096
		Yes	33,892 (26%)		30,492 (26%)	20,579 (27%)	
	Sought help any source	No	89,911 (69%)		90,279 (78%)	63,872 (83%)	< 0.0001
		Yes	39,667 (31%)		25,035 (22%)	12,650 (17%)	
	Consultation duration	mean (SD)	19 (10)		17 (9)	17 (9)	< 0.0001
	Number of problems	mean (SD)	2 (1)		2 (1)	2 (1)	< 0.0001
Consultation outcome factors	Imaging ordered	No	114,326 (88%)		102,353 (89%)	68,350 (89%)	< 0.0001
		Yes	15,252 (12%)		12,961 (11%)	8172 (11%)	
	Pathology ordered	No	100,386 (77%)		90,208 (78%)	60,039 (78%)	0.0002
		Yes	29,192 (23%)		25,106 (22%)	16,483 (22%)	
	Learning goals^a^ generated	No	83,898 (68%)		83,645 (76%)	60,082 (82%)	< 0.0001
		Yes	39,578 (32%)		26,180 (24%)	13,009 (18%)	
	Follow-up ordered	No	56,493 (44%)		52,340 (45%)	35,231 (46%)	< 0.0001
		Yes	73,085 (56%)		62,974 (55%)	41,291 (54%)	
	Referral ordered	No	107,211 (83%)		95,522 (83%)	63,393 (83%)	0.8611
		Yes	22,367 (17%)		19,792 (17%)	13,129 (17%)	
	Medication prescribed	No	54,047 (42%)		50,998 (44%)	33,314 (44%)	< 0.0001
		Yes	75,531 (58%)		64,316 (56%)	43,208 (56%)	
	Procedure performed	No	115,608 (89%)		105,838 (92%)	69,177 (90%)	< 0.0001
		Yes	13,970 (11%)		9476 (8%)	7345 (10%)	

^a^Learning goals—self-generated learning points to be pursued post-consultation (by the registrar seeking information or advice at some stage following the conclusion of the consultation)

**Table 3:** Associations of registrars’ term of training from univariate and multivariable logistic regression

			**Univariate**				**Multivariable**			
**Variable**	**Class**		**Term 1 vs Term 3**		**Term 2 vs Term 3**		**Term 1 vs Term 3**		**Term 2 vs Term 3**	
			**OR (95% CI)**	**p**	**OR (95% CI)**	**P**	**OR (95% CI)**	**p**	**OR (95% CI)**	**p**
**Registrar term level**										
**Registrar and practice variables – (registrar round level data)**										
Registrar Full Time or Part Time	Part-time		0.63 (0.54, 0.72)	< .0001	0.53 (0.46, 0.60)	< .0001	0.66 (0.57, 0.77)	< .0001	0.55 (0.48, 0.64)	< .0001
Qualified as doctor in Australia	Yes		0.90 (0.82, 1.00)	0.0501	0.96 (0.87, 1.05)	0.3293	0.90 (0.79, 1.02)	0.0926	0.99 (0.88, 1.11)	0.8493
Registrar age			0.98 (0.98, 0.99)	< .0001	0.99 (0.98, 1.00)	0.0002	0.98 (0.97, 0.99)	< .0001	0.99 (0.99, 1.00)	0.0172
Practice size	Small		1.32 (1.15, 1.51)	< .0001	1.03 (0.91, 1.18)	0.6157	1.22 (1.06, 1.41)	0.0071	0.94 (0.81, 1.08)	0.3938
Practice routinely bulk bills	Yes		1.30 (1.13, 1.50)	0.0003	1.22 (1.06, 1.41)	0.0046	0.86 (0.72, 1.03)	0.0999	0.95 (0.80, 1.13)	0.5884
Rurality	Inner regional		1.05 (0.93, 1.18)	0.4021	0.98 (0.88, 1.10)	0.7505	1.30 (1.09, 1.55)	0.0041	1.09 (0.92, 1.29)	0.3009
*Referent: Major City*	Outer regional/remote/ very remote		1.46 (1.24, 1.70)	< .0001	1.30 (1.12, 1.50)	0.0005	1.69 (1.32, 2.16)	< .0001	1.31 (1.03, 1.65)	0.0251
Geographic region	Subregion 2		1.09 (0.93, 1.28)	0.2814	1.20 (1.04, 1.38)	0.0146	0.92 (0.75, 1.13)	0.4421	1.02 (0.85, 1.24)	0.8068
*Referent: Subregion 1*	Subregion 3		0.95 (0.85, 1.05)	0.2988	0.94 (0.85, 1.03)	0.1767	0.69 (0.59, 0.81)	< .0001	0.74 (0.64, 0.86)	< .0001
	Subregion 4		1.04 (0.96, 1.13)	0.3518	1.02 (0.94, 1.09)	0.6779	1.20 (1.05, 1.37)	0.0070	0.95 (0.84, 1.07)	0.4112
	Subregion 5		3.96 (2.37, 6.63)	< .0001	3.85 (2.29, 6.47)	< .0001	2.78 (1.50, 5.16)	0.0012	2.86 (1.51, 5.40)	0.0012
	Subregion 6		2.48 (2.13, 2.88)	< .0001	2.14 (1.86, 2.46)	< .0001	3.01 (2.47, 3.66)	< .0001	2.20 (1.82, 2.66)	< .0001
Socioeconomic Index for Area-IRSD index			0.94 (0.92, 0.96)	< .0001	0.97 (0.95, 0.99)	0.0055	0.95 (0.92, 0.97)	< .0001	0.98 (0.95, 1.00)	0.1005
**Encounter Level**										
**Patient variables (adjusted for above registrar and practice variable)**										
Patient age group	0–14		0.99 (0.95, 1.03)	0.5991	1.04 (0.99, 1.09)	0.0922	0.98 (0.93, 1.03)	0.3893	1.05 (1.00, 1.10)	0.0460
*Referent: 15–34*	35–64		1.04 (1.00, 1.07)	0.0449	1.04 (1.00, 1.08)	0.0318	1.04 (1.00, 1.08)	0.0292	1.04 (1.00, 1.08)	0.0448
	65 +		1.01 (0.95, 1.07)	0.8184	1.03 (0.97, 1.10)	0.3104	1.00 (0.93, 1.06)	0.9020	1.00 (0.94, 1.07)	0.8999
Non-English speaking background	Yes		1.47 (1.31, 1.66)	< .0001	1.23 (1.09, 1.38)	0.0008	1.24 (1.10, 1.40)	0.0004	1.18 (1.04, 1.33)	0.0094
Patient/practice status	New to registrar		1.42 (1.35, 1.48)	< .0001	0.96 (0.91, 1.00)	0.0778	1.48 (1.41, 1.55)	< .0001	0.96 (0.91, 1.01)	0.1297
*Referent: Existing patient*	New to practice		1.38 (1.28, 1.47)	< .0001	0.95 (0.88, 1.02)	0.1348	1.34 (1.25, 1.44)	< .0001	0.90 (0.83, 0.96)	0.0032
**Consultation variables (adjusted for above registrar, practice and patient variables) – (consultation level data)**										
Chronic problem		Yes	0.96 (0.93, 1.00)	0.0360	0.98 (0.94, 1.01)	0.1822	0.88 (0.85, 0.92)	< .0001	0.96 (0.92, 0.99)	0.0111
Sought help any source		Yes	2.23 (2.10, 2.36)	< .0001	1.40 (1.32, 1.48)	< .0001	2.04 (1.91, 2.18)	< .0001	1.35 (1.27, 1.44)	< .0001
Consultation duration			1.02 (1.02, 1.03)	< .0001	1.01 (1.01, 1.01)	< .0001	1.02 (1.02, 1.02)	< .0001	1.01 (1.01, 1.01)	< .0001
Number of problems			1.07 (1.05, 1.10)	< .0001	0.99 (0.96, 1.01)	0.2469	1.02 (0.99, 1.05)	0.2421	0.96 (0.93, 0.98)	0.0020
**Consultation outcome variables (adjusted for above registrar, practice, patient and consultation variables) – (consultation level data)**										
Imaging ordered		Yes	1.12 (1.08, 1.16)	< .0001	1.06 (1.02, 1.10)	0.0014	0.94 (0.90, 0.98)	0.0056	0.99 (0.95, 1.04)	0.7528
Pathology ordered		Yes	1.06 (1.03, 1.09)	0.0003	1.01 (0.98, 1.04)	0.3635	0.89 (0.86, 0.92)	< .0001	1.00 (0.96, 1.03)	0.9393
Learning goals^a^ generated		Yes	2.18 (2.04, 2.32)	< .0001	1.45 (1.36, 1.53)	< .0001	1.83 (1.70, 1.97)	< .0001	1.36 (1.27, 1.45)	< .0001
Follow-up ordered		Yes	1.10 (1.05, 1.16)	< .0001	1.03 (0.98, 1.07)	0.2299	0.94 (0.89, 1.00)	0.0467	0.94 (0.89, 0.99)	0.0172
Medication prescribed		Yes	1.08 (1.04, 1.11)	< .0001	0.97 (0.94, 1.00)	0.0546	1.03 (1.00, 1.07)	0.0758	0.98 (0.95, 1.01)	0.2019
Procedure performed		Yes	1.14 (1.08, 1.20)	< .0001	0.84 (0.80, 0.89)	< .0001	1.13 (1.07, 1.20)	< .0001	0.87 (0.82, 0.92)	< .0001

^a^Learning goals—self-generated learning points to be pursued post-consultation (by the registrar seeking information or advice at some stage following the conclusion of the consultation)

Results show considerable differences in multiple variables across training, particularly between Term 1 and Term 3.

While we have not directly compared differences between Term 1 and Term 2 with differences between Term 2 and Term 3, a consideration of the overall pattern of multivariable results (effect size, p-values, direction of effect) suggests that much of the change in independent variables between early (Term 1) and later (Term 3) training and practice occurs early in training (between Terms 1 and 2).

There were several independent variables for which there were significant differences between Term 1 and Term 3, but for which the differences between Term 2 and Term 3 were of smaller effect, not reaching statistical significance, and with the direction of the association being reversed. These included registrars working in small practices (Term 1 vs Term 3 OR 1.22, Term 2 vs. Term 3 OR 0.94 (*p* = 0.007 and 0.39, respectively); registrars seeing patients that were new to them (OR 1.48 and OR 0.96 (*p* < 0.001 & 0.13); and ordering pathology (OR 0.89 and OR 1.00 (*p* < 0.001 & 0.94). For registrars seeing patients new to their practice, a similar pattern exists, though both comparisons reached statistical significance (OR 1.34 vs. OR 0.90 (*p* < 0.001 and 0.003).

For some other variables, there were differences between Terms 1 and 3, but not between Terms 2 and 3, with the values of the respective ORs suggesting much of the difference between Term 1 and 3 occurred between Term 1 and Term 2: for example, registrars working in inner regional compared to major city practice (OR 1.30 and OR 1.09 (*p* = 0.004 and 0.30), and registrars ordering imaging (OR 0.94 and OR 0.99, *p* = 0.006 and 0.75). Registrars in later terms saw significantly more patients with chronic disease, with much of this difference seeming to be between Terms 1 and 2 (OR 0.88 and OR 0.96, *p* < 0.001 and 0.011). Similarly, much of the decrease in registrars’ in-consultation seeking of information or assistance seemed to occur between Terms 1 and 2 (OR 2.04 vs. OR 1.35 *p* < 0.001 and < 0.001).

There was a lesser number of independent variables for which most difference appeared to exist between Terms 2 and 3: registrars working part-time, and the registrar organizing patient follow-up.

## Discussion

### Summary of the main findings

We found differences between Terms 1 and 3 in a range of independent variables, spanning various aspects of registrars’ practice. This suggests considerable change in the context and content of their practice as registrars progress through training. These differences were more pronounced, that is, changes were more likely to occur earlier rather than later in training. Differences were apparent especially for working in small and regional practices, and for seeing patients who were new to both registrar and the practice. Differences in seeking in-consultation information or advice, and in pathology and imaging ordering also appeared to be greater in early- than in later-training, as was exposure to chronic disease care.

Though our analyses were cross-sectional and do not document change in individual registrars’ experiences, they nevertheless strongly suggest transitions in registrar training experience are greater earlier in training. This data provides statistical insight into the magnitude and speed of transitions experienced in postgraduate training. Our research demonstrates that rapid change affecting several areas of GP registrars’ training experiences occurs earlier rather than later in training. This is evidence in support of our hypothesis that changes in demographic, clinical and educational variables are greatest earlier in training.

### Context of findings in relation to previous research

Whilst expected, change and transition have traditionally been seen as adverse periods. The management of transitions and change have featured heavily in medical educational theory and curriculum design. Traditionally, changes and transitions were viewed as problematic stages in training [10], with recommendations to alter curriculum design to align with training for the next stage, rather than addressing the transition. More recently, transitions are increasingly being viewed as learning opportunities instead of threats [20].

The transition from medical student to junior doctor, and from hospital-based work to general practice training and the attendant demands of rapid professional development, skills acquisition and tolerance of uncertainty could also be seen as entailing positive aspects. Much previous research has focused on the risk of adverse outcomes and burnout in these periods of change [2, 10, 21]. The transition into general practice training occurs in the context of GP registrars adopting a high level of independence within a new working environment and style of practice where they are expected to manage undifferentiated and complex, often chronic disease presentations [22, 23]. Experiencing a rapid change from hospital-based work to ‘the wild safari’ of GP registrar work and learning [3] can be seen as a form of ‘immersion’ into a new environment. This concept has proven effective in simulation teaching [24] and exposing medical students to work as a junior doctor [25]. Our findings, explored also in previous research [15], demonstrate that GP registrars generate less self-directed learning goals as they progress through training as seen in the differences between Terms 1 and 3 and between terms 2 and 3 (OR 1.83 and OR 1.36, *p* < 0.001 and < 0.001). This suggests that, despite the rapid transitions experienced in training, registrars develop more confidence in their clinical knowledge and practice, with less perceived need for further reflection and self-directed learning.

Conversely, rapid transitions could exacerbate negative consequences including the potential burnout associated with managing, and making decisions for [2], a high proportion of undifferentiated illnesses in general practice, especially where there is a high intolerance of clinical uncertainty [4]. Our findings show that ‘structural’ changes in training (practice size and rurality of location) are occurring concurrently with this professional and educational ‘immersion’. This may potentially amplify any consequences of this transition period, negative or otherwise, and needs to be considered when navigating this change.

A pedagogical perspective is helpful in providing a framework to consider how both personal and contextual factors influence responses to change. Using existing sociocultural educational theory helps conceptualise and clarify our findings of change and transition in an apprenticeship-like model of general practice training. This allows for a focus on how individuals can be better supported through transitions, rather than only on why this change occurs.

### Transitions and a sociocultural view of general practice training

The psychologist Lev Vygotsky describes the ‘zone of proximal development’ (ZPD) as the distance between actual and potential developmental levels. This gap in development is traversed through problem solving under the guidance of more capable peers and, ‘the more knowledgeable other’[26], in this example, the GP supervisor. The ZPD can be further differentiated into objective and subjective components [27]. The objective ZPD, applied to the apprenticeship training model, encompasses systemic functions that exist independent of the registrar, including the practices they are allocated to work in, the role of regional training organisations, the GP training curricula, and working with supervisors. The subjective ZPD describes individual capabilities in navigating change, such as resilience. This framework helps us understand how individual registrar-specific qualities and organisational components interact and influence registrars’ adaptation to new learning environments. Underpinning this in an apprenticeship-like model of training is the relationship between trainees and supervisors. Understanding the formation of this supervisory relationship [28], is essential for reducing role uncertainty and facilitating task-mastery and confidence [29].

Our findings of more rapid transitions earlier in training may be a consequence of the apprenticeship-like model of training in Australia [30]. The geographically-dispersed, more heterogeneous Australian model may facilitate the front-loaded ‘structural’ changes (such as size and rurality of practice), which comprise components of the objective ZPD, noted earlier in training. Proactive and comprehensive management of the objective ZPD would likely positively impact trainees’ experiences of transitions.

By contrast elsewhere, for example in the North American system, training is administered via state- or university-based programs with closer supervision of trainees in academic family medicine practices [6]. Patient contact and training arrangements are standardised and managed by university departments, signifying closer management of the objective ZPD for trainees.

The theory of proximal development references the instructional concepts of ‘scaffolding’ and ‘apprenticeship’ wherein more knowledgeable others, GP supervisors, help trainees formulate illness scripts and develop clinical reasoning. Our research demonstrates that the increased rate of transitions experienced earlier in training coincide with medical trainees forming more clinical questions and seeking further advice. This suggests that supervisor-led scaffolding and support is required earlier in registrar training in order to facilitate the potential of individual trainees to navigate and learn from the transitional experience, rather than be overwhelmed by it.

In the Australian GP training environment, supervisors need to be expert in providing patient care ‘by proxy’ and giving registrars support [5]. Australian GP supervisors are supported in their role by Regional Training Organisations [31]. Our findings of trainees experiencing greater clinical change earlier in training identifies the need for increased supervisor support of registrars at this time of maximal transition [32]. It may be that current emphasis on early support for registrars should be pursued even more strongly. This increased clinical support would need to be underpinned by adequate, standardised supervisor training [33], professional development and remuneration [34]. International studies have shown the need and successes of earlier support and intervention for GP trainees [35, 36].

Using established sociocultural educational theory to examine the impact of educational and behavioural changes in an apprenticeship-like model of training demonstrates where resource allocation and educational support is required, earlier in training, in order to help trainees navigate the transition to competent, independent practice.

## Conclusions

The precise impact of the quantitatively-demonstrated rapid changes early in general practice training and associated steep learning curve for trainees across a range of educational, clinical and demographic factors requires further exploration. Rapid transitions within an apprenticeship-like model of training could exacerbate already-existing impact of uncertainty in general practice and may contribute to physician burnout [2]. Pairing educational insights from established sociocultural theories of learning with our observations provides a framework for mitigating the potential negative impact of transitions. GP supervisors and training organisations, comprising the objective ZPD, need to provide trainees with additional early support. This support would help to flatten the shape of the early learning curve. Without this support, burnout and attrition rates from training programs could increase [37]. Implementing such measures may however increase demands on GP supervisors and the current apprenticeship-like Australian model of GP training.

### Further research

Further research should focus on the specific impacts that the now-identified steep learning curve has on GP trainees, supervisors, patient care and the health workforce. It remains to be explored whether particular groups of trainees are affected differently, including those training in more rural and remote areas [38] and international medical graduates training in Australia [39].

## Data Availability

The data underlying this article cannot be shared publicly due to human research ethics advice regarding requirements which protect the privacy of individuals who participated in the study.

## Abbreviations

GP: General practitioner
ReCEnT: Registrar Clinical Encounters in Training project
GEE: Generalised estimating equations
ORs: Odds ratios
ZPD: Zone of proximal development
